# Is It Necessary to Launch a School-Based Financial Literacy Curriculum? Evidence From China

**DOI:** 10.3389/fpsyg.2022.846382

**Published:** 2022-04-19

**Authors:** Xiawei Tan, Xiaoping Li, Zhineng Hu, Yongge Niu, Qianwei Ying, Yi Lu, Jiuping Xu

**Affiliations:** Business School, Sichuan University, Chengdu, China

**Keywords:** financial literacy curriculum, unobserved heterogeneity, endogenous switching, China, financial literacy

## Abstract

As starting university is a critical independence milestone for many young people, it would also be the best time to provide them with some financial education (FE). Although there have been many initiatives aimed at enhancing individual financial literacy (FL) and/or financial decision-making, meta-analyses have shown that the effectiveness of FE has been mixed. This study examined the driving forces behind the decision by college students to enroll in a targeted financial literacy curriculum (FLC) and the impact of this attendance on their FL. An endogenous switching model (ESM) was employed to account for the heterogeneity in the decision to attend or not attend the FLC and to counteract any unobservable characteristics. It was found that students with higher self-perceived FL did not prefer to attend the FLC; however, for others, FLC attendance was found to significantly boost their FL in areas such as financial knowledge (FK), financial attitude (FA), and financial behavior (FB), especially for the non-attendees under the counterfactual framework. These “non-attendees” were observed to have some characteristics (e.g., prior knowledge) that made them more financially literate regardless of attendance; however, if they had attended the FLC, they would have gained a greater FL than the attendees. As the FL of the attendees would have been much lower if they had not attended, the FLC appeared to be particularly important for the attendees, which strengthened the case for making the FLC a compulsory part of a general college education.

## 1. Introduction

As the world is becoming increasingly complex, young adults have greater financial responsibilities, which means financial literacy (FL) is necessary to successfully navigate their future (Lusardi and Wallace, 2013; OECD, 2017b). Because young adults (15–24 years old) are more malleable than adults, school-based financial education (FE) interventions are cost-efficient and a valuable approach to fostering youth FL (Frisancho, 2020; Kaiser et al., 2021). Consequently, providing FE to young people has been prioritized in many countries (OECD, 2014). Furthermore, because starting university is a critical independence milestone for many young people, it would also be the best time to provide them with FE (Gerrans, 2021). However, university FL education has generally been insufficient. For example, Fernandes et al. (2014) found that just-in-time FE was needed to avoid unreasonable financial decisions and ensure better individual choices (Thaler, 2018), the OECD (2017b) claimed that there was “an urgent need” for governments to improve all students' FL, and Lusardi and Wallace (2013) suggested that FE curricula should be part of all college and university education.

However, there is mixed evidence that FE interventions are effective. For example, Fernandes et al. (2014) found that FL interventions explained only 0.1% of the variance in financial behaviors (FBs) in 168 articles, but Miller et al. (2014)'s meta-analysis concluded that while FE interventions suffered from methodical flaws, they had a positive impact in certain areas. Kaiser et al. (2021) estimated the treatment effects of 76 FE programs on FB and FK to be 0.10 SD and 0.19 SD, respectively, and Lusardi and Mitchell (2014) found that some interventions to enhance FL worked well, but additional experimental trials were needed to discern endogeneity and establish causality.

When analyzing the impact of financial literacy curricula (FLC) on FL, researchers usually compare attendees with non-attendees. However, an FLC attendance endogeneity problem arises because FLC attendance is either voluntary or targeted toward a select group of students. For example, more financially literate students are often more likely to attend FLC; therefore, as self-selection is a possible endogeneity source, failure to account for this could overstate the true impact of any FLC. Conversely, when FLC interventions are targeted, it is more likely that fewer FL students would attend, which means that failing to account for this would also understate the true impact. Furthermore, because innate attendees with non-attendee abilities and other initial conditions are unknown, these cannot be directly controlled to identify the actual effect of FLC attendance on FL (including FK, FA, and FB). Therefore, to explicitly account for such endogeneity, simultaneous equation models are needed.

Rather than assuming that all FE interventions have a homogeneous impact on both attendees and non-attendees, this article bridges this gap by providing a micro perspective on *financial literacy curriculum* attendance and its impact on FL by adopting an *endogenous switching regression* (ESR) model. By controlling for selection bias and attendance decisions to ascertain the FE intervention effects, this study provides robust evidence on the impacts of FLC attendance on college student FL.

The remainder of this article is organized as follows. Section 2 reviews the relevant literature, Section 3 outlines the study design and the data collection, Section 4 outlines the estimation methods, Section 5 details the empirical results, Section 6 concludes the article, and Section 7 discusses the limitations.

## 2. Literature Review

**Figure 1:**
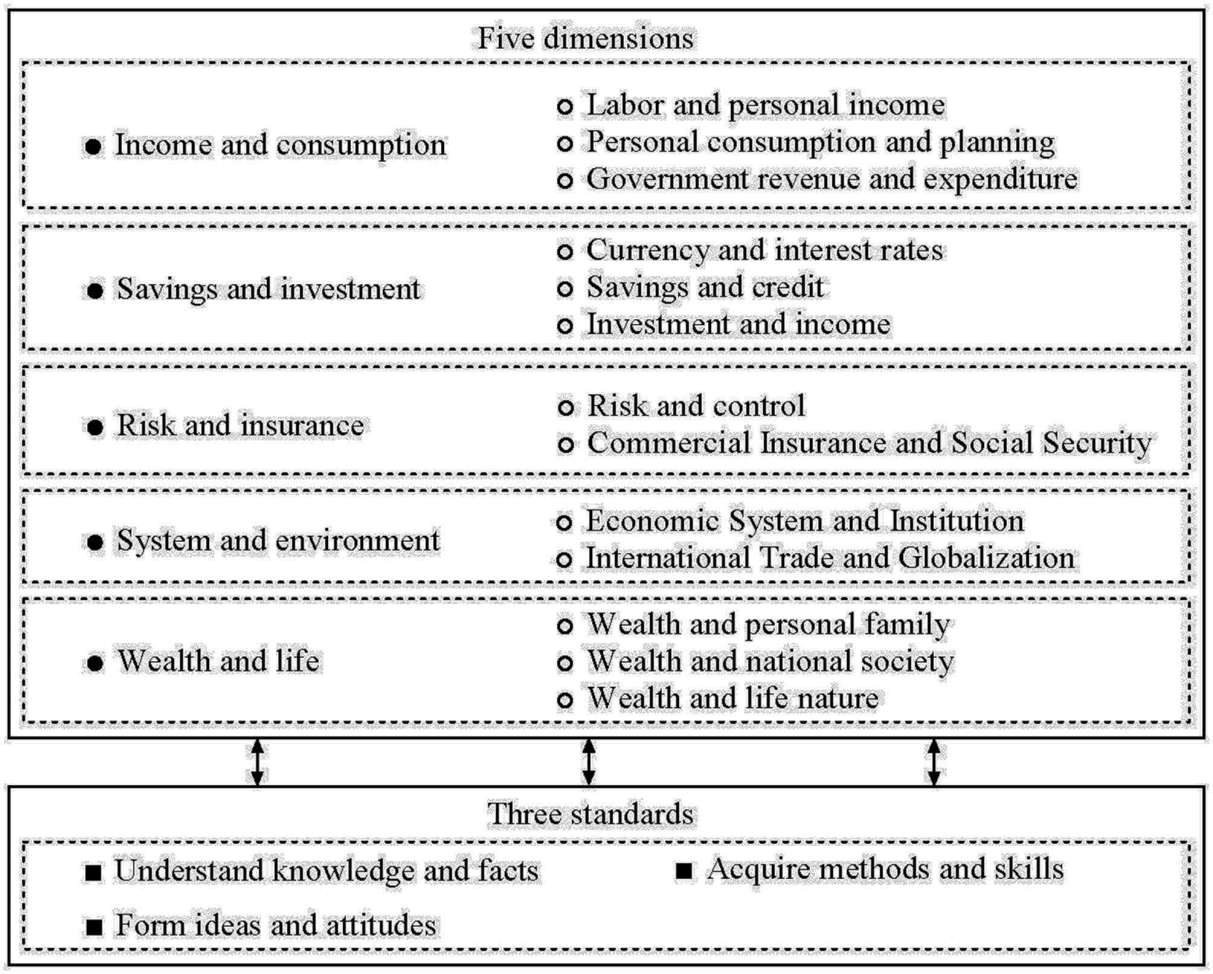
China's financial literacy (FL) education framework (China Financial Literacy Education Collaborative Innovation Center, 2018).

With the aims of promoting financial inclusion and financial stability, by 2017, over 70 countries, including most Organisation for Economic Co-operation and Development (OECD) member countries and China, had developed or implemented national strategies to enhance FE (OECD, 2015a, 2017b). The first Chinese “Financial Literacy Education Standard Framework” from kindergarten to university level was released in 2018^1^, in which the FE was divided into five dimensions and three basic elements; cognition, skills, and attitudes; which made up the “five dimensions and three standards” of the Chinese FE framework (Figure 1). Although the associated standard framework was published in China, there have been relatively few FE interventions, especially for college students.

There has been little research into the delivery and evaluation of college-level FLC, especially in developing countries. However, there has been more research in developed countries; for example, Anderson and Card (2015) assessed a convenience sample of 502 freshmen from a mid-sized US university and found that the introduction of financial responsibility courses impacted the students' decision-making, Fan and Chatterjee (2018) designed an experiment for 172 undergraduate students at a public US university, and found that investment-related FE improved their investment knowledge, and Popovich et al. (2020) assessed an online FE intervention on 97 US community college students and concluded that it had enhanced the students' financial knowledge (FK). In Europe, after a very short course (lasting less than an hour) to enhance FL levels, Brugiavini et al. (2020) found a non-negligible effect on FL in a randomized trial comprising a sample of 579 university students in Italy, with the self-assessed FL increase being larger than the actual increase in knowledge, and in Asia, Barua et al. (2018) assessed the FE provided to 632 college students using a difference-in-difference strategy, finding that the FE led to a dramatic increase in FK (improved scores of 11%) and planning (improved score by 16%). In the Oceania region, Gerrans and Heaney (2019) evaluated a personal finance semester unit delivered to 871 students at an Australian university and found objective and subjective FL increases and an additional gender effect. In developing country studies, Paraboni et al. (2020) conducted a quasi-experiment targeting 285 undergraduate business students in Brazil, finding that formal business education could improve FL. However, the FLC impact has not yet been systematically or quantitatively assessed in Chinese college students.

These previous studies indicated that targeted FE college programs could be effective in imparting FK and improving FB. One key benefit of aiming such education at college students is that an FLC could be included as a stand-alone curriculum or could be embedded within other authorized curricula. Because colleges students are a captive audience, providing FE as part of university education could reduce logistics and delivery costs and overcome participation and attendance problems.

However, few studies have explored if there are any unexpected negative results associated with FLC, i.e., do the distributional effects of these strategies intensify initial disparities if attendees are college students with baseline academic or financial performance superiorities? By adopting an ESR model, this study sought to account for FL selection bias and the discrepancies between the attendees and non-attendees. As failing to distinguish between FLC attendance causal effects and unobserved heterogeneity can also result in misleading policy implications, this study attempted to address these issues by conducting a counterfactual analysis to examine the degree to which the decision to attend or not to attend an FLC affected Chinese college student FL.

## 3. Study Design and Data Collection

A FLC designed to improve the FL of all college students was implemented by the Business School of a large-size university in southwest China in the fall 2019 semester. The FLC was a 2-credit elective course, and as there were no standardized textbooks targeting college student FL in China, experienced business professors were asked to compile the targeted textbook, which consisted of 12 chapters: independence; budgeting and savings; loans; marriage consumption; post-marital property management; debt dilemma; consumer awareness and consumer privacy; bank financial product investment; stock and fund investment; foreign exchange investment; internet finance financial management; and insurance and value preservation. After three revisions, the textbook was printed and distributed to all students who attended the FLC.

The FLC was a combination of face-to-face lectures and online financial management Massive Open Online Course (MOOC)^2^. The face-to-face instruction comprised 12 structured 3-h sessions (around 36 h in total) based on the textbook content and real financial investment and consumption cases from their teachers, colleagues, or friends, which were delivered by two senior professors (each of which covered six chapters). Every session attendance counted and was included in the final score. The online financial management MOOC courses comprised eight chapters and were delivered by a senior professor. Each student was required to study Chapter 2 and only encouraged to study all the remaining chapters^3^. Chapter 2 comprised six sessions: (a) the concept of time value (7′39″); (b) annuity (14′11″); (c) risk and reward of a single asset (11′49″); (d) risk and reward of portfolios (19′07″); (e) bond valuation (12′56″); and (f) stock valuations (18′56″). To ensure all students learned the online course, after completing the online MOOC study, students were required to complete a post-test of 18 questions within a fixed time (30 min), with the scores being included in the final grade^4^.

In the early FLC delivery stage, the school counselors distributed online questionnaires to the students enrolled at the university^5^, with the data collected from a randomized sample of students in November 2019. Students were invited *via* email to participate in the study and a second email message was sent as a reminder. Students who completed the survey became eligible to receive 25 scholarships ranging from 100 CNY (Chinese Yuan, CNY) to 500 CNY, which were awarded by a random drawing. Overall, 2,300 students were contacted, with 952 students responding to the survey, producing a response rate of 41%. Among the 952 responses, 796 were valid. After excluding those with missing data, of the 952 responses, 796 were deemed valid. Therefore, the final control group in the study comprised 796 students.

The counselors also acted as facilitators to introduce the upcoming FLC as all university students were eligible to attend. Eventually, 220 students were recruited to attend the FLC, who were then divided into two rounds; the first round from September 2019 to November 2019, and the second round from November 2019 to January 2020. Both rounds had the same teachers.

The treatment group in the study comprised 222 students, of which 219 received a grade and the remaining 3 withdrew without any academic penalty, and there were 796 students in the control group, which included eligible to enroll but had chosen not to. Therefore, the final sample comprised 1,015 students, all of whom completed a pre-course (baseline) survey; of these, the 219 treatment group students completed both the pre- and post-unit surveys.

## 4. Methodology

The simplest approach to exploring the influence of the FLC attendance on the FL level of the college students would be to include a dummy variable equal to 1 if the student attended to the FLC in the FL level equation and then use ordinary least squares (OLS). However, it was believed that this approach could result in biased estimates as it assumed that FLC attendance was exogenously determined rather than being potentially endogenous. As the decision to attend the FLC was voluntary, i.e., attendance was based on individual self-selection, students who chose to attend may have had different characteristics from those who chose not to attend, and they may also have decided to attend to gain the academic benefits. Therefore, as these unobservable characteristics could affect both the attendance and the FL levels, the FLC attendance impact could be inconsistently estimated. For example, if only the most literate or the most active college students choose to attend, and skills were failed to be controlled for, there would be an upward bias. Therefore, it was decided to employ an ESR model that accounted for both endogeneity and sample selection and allowed for complete interactions between attendance and the different FL function impact factors (Di Falco et al., 2011; Khonje et al., 2015): one FL function for attendees and another for non-attendees.

### 4.1. Decision to Enroll in the FLC

As in Khonje et al. (2015), the decision to attend the FLC was modeled in a random utility framework. Let $C_{i}^{*}$ represent the difference between the FLC attendance utility and non-attendance utility; therefore, student *i* would choose to attend the course if $C_{i}^{*}>0$. As neither utility was unobservable, they were denoted in the latent variable model with an observable component function:


(1) $$\begin{array}{l} C_{i}^{*}=Z_{i}\alpha +u_{i}\text{ with }C_{i}=\{\begin{array}{l} 1,\text{ if }C_{i}^{*}>0,\\ 0,\text{ otherwise}, \end{array} \end{array}$$

where *C*_*i*_ was a dummy variable as to whether a student *i* was the FLC attendee; 1 if the student *i* was an attendee and 0, otherwise, **α** was a parameter vector, ***Z***_*i*_ was the observable covariate vector affecting student decision-making, and *u*_*i*_ was the random error term.

Vector ***Z*** denotes the variables that impact the FLC attendance probability, with these factors being classified into different categories. Specifically, individual factors; (1) gender, grade, major, where the respondents grew up, relationship status, ranking, and whether or not attention was paid to FL; and family background; and (2) parents' education, whether the respondent was the only child, and family monthly income, were taken into account. As the FLC extension was an important way for students to obtain extra information, access to the FLC extension (both official news, social media, and others mention) was employed as a measure of information sources.

In general, women in most societies have been found to be less financially knowledgeable than men (Lusardi and Mitchell, 2011; Koh et al., 2018; Karakurum-Ozdemir et al., 2019; Swiecka et al., 2020); therefore, it was assumed that the men attending the FLC would have a higher level FL. As college student's FL scores have been found to be significantly associated with their year of study from 1st year to Master's level (Jorgensen, 2007), it was also assumed that the students of the Master's level may have a lesser preference to attend the FLC.

Social interaction theory points out that social interactions with siblings provide a socialized development environment for young adults, which is beneficial to their psychological development and core literacy formation (Dunn, 1988). Therefore, it was hypothesized that college students from one-child families would have lower financial decision-making and would tend not to choose to attend the FLC. Because educated people have greater opportunities to take financial management-related courses, they also have higher levels of FL (Lusardi and Mitchell, 2011, 2014). Social learning theory (Bandura and Walters, 1977) suggests that social behavior is learned by observing and imitating the behavior of others; therefore, it was assumed that parental education levels would affect the students' FL.

Cui et al. (2019) found that people living in urban regions had better FL than those living in rural areas. The social learning process also suggests that having a partner could provide greater opportunities to share and gather experiences, which could improve FL. Therefore, relationship (marital) status was seen to be closely related to an individual's FL. Students' grade rankings also positively impact FL. Al-Bahrani et al. (2020) detected that math ability and relative education could foster college students' FL; therefore, students who were higher ranked may prefer to attend an FLC.

Douissa (2020)'s research indicated that college students who exhibited a lower level of FL did not take an FLC and had lower family incomes, and Ergün (2018) found that people whose parents had high-level incomes were more financially literate; therefore, it was assumed that students with high-level income parents were more likely to attend an FLC. Ergün (2018) also explored the impact of “getting financial information about financial issues” on college student's FL, and found that the coefficients for the above dummy variables were all significant; therefore, it was assumed that those who had paid attention to FL would choose to attend an FLC.

Speculating that the outcome variable was a linear function, the college students' FL (comprising of FK, FB, and FA) impact was expressed as:


(2) $$\begin{array}{l} Y_{i}=X_{i}\gamma +\delta C_{i}+\upsilon_{i}, \end{array}$$

where *Y*_*i*_ was the students' FL (FK, FB, or FA), ***X***_*i*_ was a series of other variables that impacted the students' FL (FK, FB, or FA), *C*_*i*_ was a variable for attendance as defined before, **γ** and **δ** were the parameters to be estimated, and υ_*i*_ was an error item.

The impact of attendance on FL (FK, FB, or FA) was measured using an **δ** estimation. However, if **δ** were to precisely measure the FE attendance on FL (FK, FB, or FA), the students needed to be randomly assigned to either the attendance or non-attendance groups.

### 4.2. FLC Attendance Impact Evaluation

#### 4.2.1. Endogenous Switching Regression

The major focus of this study was to explore the influence of attending an FLC on FL (comprising FK, FB, and FA), which was assessed from the average treatment effect on the treated (ATT), which calculated the average differences in the FL of the attendees being treated with the FLC with those who did not attend. Methods such as the propensity matching method disregard unobservable variables such as student interest and their FLC perceptions and presume the socio-demographic coefficient to be the same for both attendees and non-attendees, which does not align with reality (Ma and Abdulai, 2016). Therefore, this study accounted for the endogeneity of the take-up decision by estimating a simultaneous equations model for FLC attendance and FL, with the endogenous switching using full-information maximum likelihood (FIML) (Di Falco et al., 2011). The endogenous switching FL regression model in which the students faced two regimes (to attend or not to attend) was defined as follows:


(3) $$\begin{array}{l} \text{Regime}\;1\;:\;Y_{1i}=X_{1i}\beta_{1}+\varepsilon_{1i},\text{ if }C_{i}=1,\\ \text{Regime}\;2\;:\;Y_{0i}=X_{0i}\beta_{0}+\varepsilon_{0i},\text{ if }C_{i}=0, \end{array}$$

where ***X***_1*i*_ and ***X***_0*i*_ were exogenous covariates, **β**_1_ and **β**_0_ were the parameter vectors to be estimated, and ε_1*i*_ and ε_0*i*_ were random disturbance terms.

The estimation for **β**_**0**_ and **β**_**1**_ could be biased because the conditional error item expectations (η_1_ and η_0_) were non-zero (Shiferaw et al., 2014). The error terms in Equations (1) and (3) were presumed to be trivariate normally distributed with a mean vector and a covariance matrix:


(4) $$\begin{array}{l} \Sigma =\text{Cov}\left(\eta ,\varepsilon_{1},\varepsilon_{0}\right)=\left[\begin{array}{ccc} \sigma_{\eta }^{2}\; & \sigma_{\eta 1}\; & \sigma_{\eta 0}\\ \sigma_{\eta 1}\; & \sigma_{1}^{2}\; & \cdot \\ \sigma_{\eta 0}\; & \cdot \; & \sigma_{0}^{2} \end{array}\right], \end{array}$$

where $\sigma_{\eta }^{2}$ = Var (η), $\sigma_{1}^{2}$ = Var (ε_1_), $\sigma_{0}^{2}$ = Var (ε_0_), σ_η1_ = Cov (η, ε_1_), and σ_η0_ = Cov (η, ε_0_).

Assuming that $\sigma_{\eta }^{2}$ equaled 1, as *Y*_1*i*_ and *Y*_0*i*_ were never simultaneously observed, the covariance between ε_1*i*_ and ε_0*i*_ was not defined. Because *u*_*i*_ [in Equation (1)] was correlated with ε_1*i*_ and ε_0*i*_ [Equation (3)], the expected values for the ε_1_ and ε_0_ conditioning on the sample selection were non-zero (Asfaw et al., 2012).


(5) $$\begin{array}{l} E\left(\varepsilon_{1i}|C_{i}=1\right)=\sigma_{1\eta }\frac{\phi \left(Z_{i}\alpha \right)}{\Phi \left(Z_{i}\alpha \right)}\;\equiv \;\sigma_{1\eta }\lambda_{1i},\\ E\left(\varepsilon_{0i}|C_{i}=0\right)=-\sigma_{0\eta }\frac{\phi \left(Z_{i}\alpha \right)}{1-\Phi \left(Z_{i}\alpha \right)}\;\equiv \;\sigma_{0\eta }\lambda_{0i}, \end{array}$$

where ϕ(***Z***_*i*_**α**) and Φ(***Z***_*i*_**α**) denoted the density function and the cumulative density function for the standard normal distribution with ***Z***_*i*_**α** as the variables, $\frac{\phi \left(Z_{i}\alpha \right)}{\Phi \left(Z_{i}\alpha \right)}$ (denoted λ_1*i*_) and $-\frac{\phi \left(Z_{i}\alpha \right)}{1-\Phi \left(Z_{i}\alpha \right)}$ (denoted λ_0*i*_) representing the selection biases resulting from the unobservable variables, which were the inverse Mills ratios (IMR), i.e., if σ_1η_ and σ_0η_ were significantly non-zero, it was necessary to correct the sample selection bias caused by the unobservable variables.

The above ESR framework was used to estimate the average treatment effect of the treated (ATT) and untreated (ATU) by comparing the expected values for the attendee and non-attendee outcomes in actual and counterfactual scenarios. Following Di Falco et al. (2011) and Shiferaw et al. (2014), the ATT and ATU were calculated as follows:

Attendees with attendance (observed in the sample)


(6) $$\begin{array}{l} E\left(Y_{1i}|C_{i}=1\right)=X_{1i}\beta_{1}+\sigma_{1\eta }\lambda_{1i}. \end{array}$$

Attendees had decided not to attend (counterfactual)


(7) $$\begin{array}{l} E\left(Y_{0i}|C_{i}=1\right)=X_{1i}\beta_{0}+\sigma_{0\eta }\lambda_{1i}. \end{array}$$

Non-attendees had decided to attend (counterfactual)


(8) $$\begin{array}{l} E\left(Y_{0i}|C_{i}=0\right)=X_{0i}\beta_{1}+\sigma_{1\eta }\lambda_{0i}. \end{array}$$

Non-attendees without attendance (observed in the sample)


(9) $$\begin{array}{l} E\left(Y_{1i}|C_{i}=0\right)=X_{0i}\beta_{0}+\sigma_{0\eta }\lambda_{0i}. \end{array}$$

The ATT is the difference between Equations (6) and (7):


(10) $$\begin{array}{l} \text{ATT}=\;\left(Y_{1i}|C_{i}=1\right)-\left(Y_{0i}|C_{i}=1\right),\\ \;=\;X_{1i}\left(\beta_{1}\;-\beta_{0}\right)+\lambda_{1i}\left(\sigma_{1\eta }\;-\sigma_{0\eta }\right). \end{array}$$

The ATU is the difference between Equations (8) and (9):


(11) $$\begin{array}{l} \text{ATU}=\;\left(Y_{1i}|C_{i}=0\right)-\left(Y_{0i}|C_{i}=0\right),\\ \;=\;X_{0i}\left(\beta_{1}\;-\beta_{0}\right)+\lambda_{0i}\left(\sigma_{1\eta }\;-\sigma_{0\eta }\right). \end{array}$$

#### 4.2.2. FL Measurement

While there is no generally accepted FL definition, the G20 leaders (OECD, 2017a) and the OECD (2016) have defined FL as “a combination of the awareness, knowledge, skills, attitude [sic] and behaviors necessary to make sound financial decisions and ultimately achieve individual financial well-being,” which tends to indicate that FL is more than just FK as it also embraces a good FA and informed FB. This study adopted this FL definition (OECD, 2016), and following this definition, the OECD (2015b) questionnaire was used for the data collection.

To explore the overall FL (FK, FA, and FB), the online instrument was based on the OECD (2015b) toolkit, which has repeatedly shown good reliability and validity on Chinese samples (OECD, 2017a). The FK was measured using eight questions (Appendix 7); time value of money, interest paid on a loan, interest plus principal, compound interest, risk and return, the definition of inflation, diversification, and bond knowledge; with each correct answer was given a score of one; therefore, the total FK score was eight. The FK score was computed as the number of correct responses to the FK questions, with each correct answer scoring one and all others scoring zero; therefore, the total score ranged between 0 and 8.

Financial behavior was evaluated through responses to questions that focused on how the respondents dealt with money in their daily lives, for which nine items were adopted; timely bill payment, keeping watch of financial affairs, long-term financial goal setting, borrowing to make ends meet, choosing products, responsible and has a household budget^6^, active saving, and choosing financial products. The behavior score was computed as a count of the number of “financially savvy” behaviors, with the score ranging from 0 and 8 (Appendix 7).

The survey had three FA statements (Appendix 7) to gauge the students' attitudes toward money and planning for the future; spending rather than saving, living for today, and money is there to be spent; with the attitude score, which ranged from 1 to 5, calculated by adding up the values of the three statements and then dividing by three, with the total scores ranging from 1 to 5.

Because both the OECD (2015b) and Agarwalla et al. (2015) added up these three functionally independent FL dimension scores to measure FL, therefore, this study also adopted this method to gauge the Chinese college students' FL, with the highest possible score being 21 and the lowest being 1.

## 5. Results and Discussion

### 5.1. Sample Characteristics

Because the 2019 semester FLC was an elective, it was important to carefully consider any possible self-selection effects when assessing the outcomes attributable to the FLC enrolment. To identify these outcomes, those who attended the FLC were compared with a control group drawn from students who were otherwise eligible to attend but chose not to attend.

Table 1 shows the comparison of the observable characteristics between the treatment and control groups, the information for which was collected from the survey. Except for the respondents' general grade ranking and whether the respondent was an only child, there were significant differences in almost all of the other characteristics. Compared to the non-attendees, the attendees showed better FL (including FK, FA, and FB), and women and 2nd-year undergraduates were more likely to attend the FLC. The variables, such as where the respondents grew up, relationship status, major, parents' education, family income, attention to FL, and the information sources were also different in the two groups.

**Table 1:** Respondent summary.

	**Attendees**	**Non-attendees**	**Attendees-Non-attendees**	**p-value difference**
Dependent variables				
FL	15.790	13.178	2.612	<0.001
FK	6.210	5.384	0.826	<0.001
FB	6.256	4.907	1.349	<0.001
FA	3.324	2.887	0.438	<0.001
Explanatory variable				
Gender	0.680	0.494	0.187	<0.001
Grade				
1^*st*^ year	0.041	0.231	−0.190	<0.001
2^*nd*^ year	0.813	0.480	0.333	<0.001
3^*rd*^ year	0.055	0.258	−0.203	<0.001
4^*th*^ year	0.087	0.005	0.082	<0.001
Postgraduate	0.005	0.026	−0.022	0.050
City	0.607	0.695	−0.087	0.014
Relationship	0.228	0.423	−0.195	<0.001
OnlyChild	0.438	0.469	−0.030	0.427
Major				
Economics and management	0.330	0.410	−0.080	0.03
LiberalArts	0.237	0.422	−0.185	<0.001
Science	0.128	0.078	0.050	0.021
Engineering	0.219	0.080	0.139	<0.001
Medical	0.087	0.010	0.077	
Father's education				
≤Middle school	0.350	0.510	−0.160	<0.001
HighSchool	0.333	0.160	0.174	<0.001
≥Bachelor	0.320	0.334	−0.015	0.686
Mother's education				
≤Middle school	0.430	0.550	−0.120	0.002
HighSchool	0.356	0.165	0.192	<0.001
≥Bachelor	0.210	0.284	−0.074	0.029
Ranking				
First	0.150	0.150	0.000	0.961
Second	0.196	0.188	0.008	0.792
Third	0.274	0.280	−0.006	0.857
Last	0.379	0.379	0.000	0.991
Family income				
Low	0.190	0.520	−0.330	<0.001
Medium	0.297	0.268	0.029	0.391
High	0.215	0.099	0.115	<0.001
VeryHigh	0.100	0.075	0.025	0.228
DK	0.201	0.036	0.164	
Attention to FL	0.411	0.915		<0.001
Information sources				
News	0.849	0.759	0.091	0.004
Othersmention	0.616	0.535	0.081	0.032
Media	0.589	0.590	−0.001	0.970

*Sample size: 1,015. The number of attendees were 219 and the non-attendees were 796*.

To give a complete description of how the scores fluctuated over time, the M, SD, min, and max scores for the change scores were also provided for the attending and non-attending groups (Appendix 7). The results showed that attending groups experienced a dramatic improvement in their FL level after completing the FLC, with a magnitude of around 25.14%. It was assumed that the FL level of the non-attending groups would not have varied over a relatively short period (less than 1 year).

### 5.2. FLC Participation Determinants

Logistic regression was performed to evaluate the estimated FLC attendance parameters (Tables 2–**4**). For all specifications, the same variables had statistically similar effects on attendance. Generally, eight variables were found to be significant in explaining the FLC attendance: gender, grade, where the respondents grew up, relationship status, major, parents' education, family income, and attention to FL.

**Table 2:** Endogenous switching regression (ESR) estimates for student's financial literacy (FL).

	**(1)**	**(2)**	**(3)**	**(4)**
**Model**	**OLS**	**Endogenous switching regression**		
			**Attendance = 1**	**Attendance = 0**
			**Attendees**	**Non-attendees**
**Dependent variable**	**FL**	**Attendance**	**FL**	**FL**
FE	3.023^***^			
	(0.191)			
Gender	0.065	0.544^***^	−0.369	0.033
	(0.122)	(0.127)	(0.317)	(0.136)
Grade				
2^*nd*^ year	−0.135	1.411^***^	−1.675^***^	−0.144
	(0.167)	(0.220)	(0.640)	(0.191)
3^*rd*^ year	−0.405^**^	0.320	−1.771^**^	−0.333^*^
	(0.191)	(0.273)	(0.765)	(0.193)
4^*th*^ year	−1.138^**^	3.047^***^	−2.794^***^	−2.231^**^
	(0.442)	(0.451)	(0.843)	(0.980)
Postgraduate	0.772^*^	−0.041	4.147^**^	0.725^*^
	(0.432)	(0.538)	(1.696)	(0.431)
City	0.835^***^	−0.379^***^	1.538^***^	0.427^**^
	(0.143)	(0.140)	(0.306)	(0.167)
Relationship	0.139	−0.428^***^	0.372	0.162
	(0.127)	(0.130)	(0.306)	(0.138)
OnlyChild	−0.048	−0.064	0.424	−0.045
	(0.120)	(0.123)	(0.272)	(0.132)
Major				
LiberalArts	0.210	−0.386^***^	0.213	0.217
	(0.130)	(0.138)	(0.321)	(0.140)
Engineering	0.388^*^	0.709^***^	−0.422	0.381
	(0.203)	(0.172)	(0.364)	(0.258)
Father's education				
HighSchoolF	0.342^*^	0.580^***^	−0.026	0.325
	(0.194)	(0.182)	(0.373)	(0.233)
BachelorF	0.084	0.212	−0.011	0.170
	(0.215)	(0.221)	(0.444)	(0.241)
Mother's education				
HighSchoolM	−0.168	0.035	−0.242	−0.027
	(0.196)	(0.184)	(0.359)	(0.229)
BachelorM	0.138	−0.531^**^	0.521	0.230
	(0.219)	(0.229)	(0.480)	(0.245)
Ranking				
Second	−0.281	−0.049	−0.573	−0.337
	(0.205)	(0.202)	(0.430)	(0.226)
Third	−0.463^**^	−0.056	−1.124^***^	−0.307
	(0.192)	(0.190)	(0.425)	(0.210)
Last	−0.638 ^***^	−0.070	−1.799^***^	−0.287
	(0.183)	(0.181)	(0.412)	(0.200)
Family income				
Medium	0.032	0.427^***^	0.021	0.011
	(0.147)	(0.145)	(0.306)	(0.164)
High	0.256	0.772^***^	−0.798^**^	0.643^**^
	(0.201)	(0.195)	(0.376)	(0.242)
VeryHigh	0.470^**^	0.748^***^	0.011	0.505^*^
	(0.234)	(0.227)	(0.459)	(0.269)
Attention to FL	0.647^***^	−1.542^***^	1.213^***^	1.463
	(0.180)	(0.139)	(0.389)	(0.283)
Information sources				
news		0.458^***^		
		(0.143)		
Othersmention		0.299^**^		
		(0.116)		
Media		−0.103		
		(0.119)		
Constant	12.234^***^	−1.445^***^	18.374^***^	11.470^***^
	(0.308)	(0.339)	(0.909)	(0.378)
σ_*i*_			1.960^***^	1.827^***^
			(0.157)	(0.046)
ρ_*j*_			−0.762^***^	−0.050
			(0.105)	(0.166)

*Sample size: 1,015. Robust standard errors in parentheses. σ_i_ denoted the square-root of the variance of the error terms η_ji_ in the outcome Equation (3); ρ_j_ denotes the correlation coefficient between the error term ε_i_ of the selection Equation (1) and the error term η_ji_ of the outcome Equation (3)*.

*** *Significant at 1%*;

** *Significant at 5%*;

* *significant at 10%*.

Gender has been assumed to significantly affect the possibility of college students participating in an FLC, with women being more willing to participate in the FLC than men. Positive correlations have been found between an individual's objective FL and their subjective FL. Gignac (2022) found that people typically had some perceptions of their FL, with the degree of this perception being nearly equal to their objective FL. Therefore, it was assumed that as female college students would realize that they had inferior FL, they would be more willing to participate in the FLC to improve their FL.

Student's grade was also assumed to significantly affect the possibility of college students participating in the FLC; for example, Jorgensen (2007) found that students' FL scores gradually boosted from first year to Masters. Contrary to their counterparts (the 1st-year students), the 2nd year and the 4th-year students were found to have a higher interest in the course, which indicated that the 2nd-year students and the 4th-year students were more likely to join the FLC.

The student's family income significantly affected FLC attendance. Compared to the students with relatively low family incomes, students with higher family incomes were more likely to attend the FLC. *Consumer socialization theory* (Moschis and Churchill, 1978) claims that school, mass media, family, and peers influence the acquisition by young individuals of knowledge and skills related to their later adult roles. Mancebón et al. (2019) found that the importance of the family influenced the FL of 15-year-old Spanish students, Moreno-Herrero et al. (2018) showed that being exposed to financial products and discussing money matters with parents improved student FL, and Zhu (2019) found that parental financial socialization positively influenced adolescent FBs. Therefore, it was surmised that students with higher family incomes were more influenced by financially literate parents that have some insight into the importance of FE, and therefore, they were more likely to attend the FLC.

Students who grew up in a city were less likely to participate in the FLC than students who grew up in rural areas. As people living in urban regions have generally been found to have better FL (Cui et al., 2019), their need to improve their FL would be relatively weak; therefore, they would be less likely to participate in an FLC to improve their FL.

Single college students were more likely to participate in the FLC than non-single students. Kiliyanni and Sivaraman (2016) found that non-single respondents were more financially literate than single respondents. Therefore, single college students who perceived that they had weak FL would spend more time and energy on the elective FLC courses.

Those who majored in science were more likely to participate in the FLC than students who majored in finance. Ergün (2018) found that business majors were more knowledgeable about personal finance than non-business majors; therefore, it was surmised that non-business major students, who probably had relatively lower FL, would be more willing to improve their FL through an FLC.

Mother's education also significantly affected the likelihood of FLC attendance, with students with mothers who had a bachelor's degree or above and above being found to be less likely to attend an FLC than those whose mothers only had a junior school education or below. Lusardi and Mitchell (2011) found FL was significantly and positively correlated with parental education (and in particular, that of mothers) in students aged 23–28. Therefore, as students with mothers with only a junior school education or below may believe that their FL to be inferior, they would be more likely to attend an FLC to improve their FL.

Father's education also significantly affected the likelihood of FLC attendance, with students whose fathers had a high school degree being found to be more likely to attend an FLC than those whose mothers only had a junior school education or below. Jorgensen (2007) found that students who were influenced by their parents' finances (education or status) scored higher on FL. Although the respondents' fathers' education was comparatively high, their father's FE was not necessarily high. Similarly, as students with fathers who had a high school diploma might believe that their FL was not high, they would be more likely to attend an FLC to improve their FL.

Compared to students who have paid attention to their FL, those who had not focused on FK would probably be more likely to attend an FLC. Ergün (2018) found that college students who had paid attention to FL exhibited higher FL levels; therefore, it was considered likely that those who had not focused on FK would be more likely to want to improve their FL by attending an FLC. It was also surmised that information on the FLC extension could play an important role in determining the students' decisions to attend.

In a word, Chinese college students with higher FL were assumed to be more likely to attend the FLC. Furthermore, Gignac (2022) found that people's perceptions of their FL (subjective FL) were nearly equal across the spectrum to their objective FL; therefore, it was possible that students with high self-perceived FL would not choose to attend an FLC.

### 5.3. Financial Literacy

The estimates for the determinants for attendance and the impact of attendance on FL and its three components are presented in Tables 2–**5**. The first column presents the OLS estimation without the switching and with a dummy variable equal to 1 if the college student decided to attend to the FLC and 0, otherwise, and the second to fourth columns, respectively, document the estimated coefficients of selection in Equation (1) for attending or not attending the FLC and for the FL (FK, FA, and FB) Equation (3) for college students that did and did not attend to the FLC.

The endogeneity of the attendance decision was accounted for by estimating a simultaneous equation model for FLC participation and FL with endogenous switching by full information maximum likelihood (FIML). For the model to be identified, it was necessary to use as exclusion restrictions. Therefore, as selection instruments, not only those automatically generated by the nonlinearity of the selection model (Eq. 1) but also other variables that directly affect the selection variable but not the outcome variable.

As in Di Falco et al. (2011), the selection instruments used in the FL function were the variables related to the FLC information sources; college financial news, mentioned by others, social media, if received, and especially information on the FLC. We verified the admissibility of the instruments by conducting a simple falsification test. A variable is an effective selection instrument if it would affect the FLC attendance decision of all of the students, and it would not affect the FL level of the non-attending students. Table 14 in the Appendix shows that the FLC information sources were valid selection instruments as they were found to be jointly statistically significant drivers of the decision to attend or not to the FLC but not statistically significant drivers for the FL (FK, FB, and FA) level of the students that did not attend.

When estimating the FLC effect on college students' FL using the OLS, the coefficient was significantly positive, which indicated that FLC attendance significantly and positively impacted the college students' FL (Table 2). However, the OLS estimation assumed that the FLC attendance was exogenous, which means there may have been biased and inconsistent estimates. In addition, OLS estimates do not explicitly account for the potential structural differences between the FL function of college students that attended the FLC and the FL function of college students that did not attend.

An interesting finding was the signs and significance of the covariance terms for the attendees [Tables 2–**5**, columns (3)]. As the statistically significant covariance estimate for the attendees indicated that there was self-selection, the FLC attendance of the FLC may not have had the same effect on the non-attendees if they had chosen to attend (Lokshin and Sajaia, 2004). Furthermore, as the negative sign for ρ_*j*_ of the attendees indicated a positive selection bias, this suggested that students with above-average FL (FK, FA, and FB) had a higher probability of attending the FLC. Therefore, comparative advantage tended to play a critical role in the determination of FL (FK, FA, and FB), and attendance decisions. In contrast, The statistically insignificant covariance terms for the non-attendees suggested that in the absence of the FLC, there would be no significant difference in the average behavior of the two groups of students resulting from unobservable factors.

The differences in the coefficients for the FL equation between the attending group and the non-attending group implied the presence of heterogeneity in the sample [Table 2, columns (3) and (4)]. The FL level function of the attending group was significantly different (at a 1% statistical level) from the FL level function of the non-attending groups. It was expected that grade, where they grew up, ranking, family monthly income, and attention paid to FK would be significantly associated with changes in the FL; however, grade and family monthly income seemed to more significantly affect the FL level of the non-attending group.

Another interesting difference between the attendees and non-attendees was the impact of household monthly income. Differently from the existing literature, the impact of household monthly income was analyzed for the two different groups, and it was found that while high household monthly income negatively affected the FL of the attendees, it positively impacted the FL of the non-attendees, which indicated that an increase in household monthly income would strengthen the non-attendees' FL level.

Table 3 shows the college student FL levels under factual and counterfactual conditions. Cells (*a*_1_) and (*b*_1_) represent the college students' FL observed in the sample. The attending group's expected FL score was about 15.784 points, while that of the non-attending group was about 13.178 points. However, this simple comparison may be misleading and prompt researchers to conclude that the average. In other words, the attendees would score about 2.606 (or around 19.8%) higher than that of the non-attendees.

**Table 3:** Average expected FL; treatment and heterogeneity effects.

**Sub-samples**	**Decision stage**		**Treatment effects**
	**To attend**	**Not to attend**	
College students that attended	(*a*_1_) 15.784	(*c*_1_) 12.249	TT = 3.535^***^
	(0.086)	(0.065)	(0.108)
College students that did not attend	(*d*_1_) 18.831	(*b*_1_) 13.178	TU = 5.653^***^
	(0.054)	(0.023)	(0.059)
Heterogeneity effects	BH_1_ = −3.047^***^	BH_2_ = −0.929^***^	TH = −2.118^***^
	(0.113)	(0.056)	(0.104)

*** *Significant at 1%*.

The last column in Table 3 shows the effect of participating in the FLC on the students' FL. In the counterfactual situation (*c*_1_), if the attendees did not attend, the FL score would be reduced by about 3.535 (i.e., about 22.4%). In the counterfactual situation (*d*_1_), if the non-attendees attended, their FL score would increase by 5.653 (i.e., about 42.9%). These results indicated that the FLC attendance would significantly improve the Chinese college students' FL; however, the transitional heterogeneity effect was negative, i.e., the FLC impact on the attendees was significantly less than that of the non-attendees.

The last row of Table 3 adjusts the potential heterogeneity in the sample and indicated that in the counterfactual situation (*c*_1_), the attendees' FL level would be significantly lower than the non-attendees. Finally, in the counterfactual situation (*d*_1_), if the non-attendees had participated in the FLC, their FL level would have far exceeded that of the actual attendees'. This result highlights that, regardless of whether or not a student attended the FLC, there were some important sources of heterogeneity that make the non-attendees appear to be “better learners” than the attendees.

### 5.4. Financial Knowledge

When estimating the FLC effect on the college students' FL using the OLS, the coefficient was significantly positive, which indicated that the FLC attendance significantly and positively impacted the college students' FK (Table 4).

**Table 4:** Endogenous switching regression estimates for student's financial knowledge (FK).

	**(1)**	**(2)**	**(3)**	**(4)**
**Model**	**OLS**	**Endogenous switching regression**		
			**Attendance = 1**	**Attendance = 0**
			**Attendees**	**Non-attendees**
**Dependent variable**	**FK**	**Attendance**	**FK**	**FK**
FE	0.875^***^			
	(0.122)			
Gender	0.117	0.518^***^	−0.521^**^	0.120
	(0.078)	(0.122)	(0.249)	(0.089)
Grade				
2^*nd*^ year	0.101^*^	1.385^***^	−1.277^***^	0.121
	(0.107)	(0.213)	(0.450)	(0.132)
3^*rd*^ year	−0.032^***^	0.326	−0.369^**^	−0.010
	(0.122)	(0.266)	(0.519)	(0.123)
4^*th*^ year	−0.475	2.571^***^	−2.229^***^	−1.560^**^
	(0.282)	(0.458)	(0.658)	(0.627)
Postgraduate	0.245	−0.047	1.890^*^	0.184
	(0.276)	(0.534)	(1.094)	(0.276)
City	0.242	−0.404^***^	0.773^***^	0.099
	(0.091)	(0.137)	(0.223)	(0.108)
Relationship	0.078	−0.406^***^	0.515^**^	0.087
	(0.081)	(0.126)	(0.221)	(0.089)
OnlyChild	0.014	−0.046	0.556^**^	−0.074
	(0.077)	(0.119)	(0.198)	(0.084)
Major				
LiberalArts	0.011	−0.356^**^	0.334	0.027
	(0.083)	(0.134)	(0.249)	(0.090)
Engineering	0.137	0.650^***^	−0.459	0.048
	(0.130)	(0.170)	(0.297)	(0.167)
Father's education				
HighSchoolF	−0.107^***^	0.565^***^	−0.631^**^	−0.012
	(0.124)	(0.175)	(0.287)	(0.150)
BachelorF	−0.095	0.336	−0.290	−0.067
	(0.137)	(0.219)	(0.324)	(0.155)
Mother's education				
HighSchoolM	−0.030	0.023	−0.038	0.059
	(0.125)	(0.175)	(0.267)	(0.147)
BachelorM	0.098	−0.608^**^	0.659^*^	0.112
	(0.140)	(0.223)	(0.347)	(0.159)
Ranking				
Second	0.005	−0.063	−0.176	0.008
	(0.131)	(0.193)	(0.312)	(0.144)
Third	−0.013	−0.052	−0.259	0.043
	(0.122)	(0.183)	(0.303)	(0.134)
Last	−0.177	−0.013	−0.498^*^	−0.063
	(0.117)	(0.177)	(0.298)	(0.128)
Family income				
Medium	−0.066	0.409^***^	−0.333	−0.037
	(0.094)	(0.140)	(0.227)	(0.106)
High	−0.034	0.738^***^	−0.716^**^	0.110
	(0.128)	(0.188)	(0.288)	(0.156)
VeryHigh	0.226	0.725^***^	−0.394	0.266
	(0.149)	(0.217)	(0.344)	(0.174)
Attention to FL	0.035	−1.465^***^	1.594^***^	−0.139
	(0.115)	(0.138)	(0.334)	(0.197)
Information sources				
News		0.286^*^		
		(0.151)		
Othersmention		0.314^***^		
		(0.105)		
Media		−0.237^**^		
		(0.106)		
Constant	5.127	−1.267^***^	8.136^***^	5.274^***^
	(0.197)	(0.330)	(0.703)	(0.254)
σ_*i*_			1.544^***^	1.168^***^
			(0.169)	(0.029)
ρ_*j*_			−0.918^**^	−0.052
			(0.083)	(0.215)

*Sample size: 1,015. Robust standard errors in parentheses. σ_i_ denoted the square-root of the variance of the error terms η_ji_ in the outcome Equation (3); ρ_j_ denotes the correlation coefficient between the error term ε_i_ of the selection Equation (1) and the error term η_ji_ of the outcome Equation (3)*.

*** *Significant at 1%*;

** *Significant at 5%*;

* *Significant at 10%*.

The FK level function of the attending group was significantly different (at a 1% statistical level) from the FK level function of the non-attending group [Table 4, columns (3) and (4)]. It was expected that grade, hometown, relationship status, father's education, mother's education, ranking, family monthly income, and attention paid to FK were significantly associated with changes in the FK; however, grade seemed to more significantly affect the FK level of the non-attending group.

The ESR results showed that for the attending group, the high family monthly income had a negative impact on the attendees' FK at a 5% significance level, i.e., attendees with higher family monthly income showed a significantly lower FK level than that of the average college students in the sample. In contrast, high family monthly income was not found to affect the FK of the attendees.

Table 5 shows the FK level of the college students under the factual and counterfactual conditions. Cells (*a*_2_) and (*b*_2_) represent the college students' FK observed in the sample. The attending group's expected FK score was about 6.210 points, while that of the non-attending group was about 5.384 points. However, this simple comparison may be misleading and prompt researchers to conclude that on average, the attendees' FK score would be about 0.826 (around 15.3%) higher than that of the non-attendees.

**Table 5:** Average expected FK; treatment and heterogeneity effects.

**Sub-samples**	**Decision stage**		**Treatment effects**
	**To attend**	**Not to attend**	
College students that attended	(*a*_2_) 6.210	(*c*_2_) 5.330	TT = 0.880^***^
	(0.040)	(0.035)	(0.053)
College students that did not attend	(*d*_2_) 9.569	(*b*_2_) 5.384	TU = 4.184^***^
	(0.030)	(0.007)	(0.031)
Heterogeneity effects	BH_1_ = −3.359^***^	BH_2_ = −0.055^***^	TH = −3.304^***^
	(0.061)	(0.023)	(0.058)

*** *Significant at 1%*.

The last column in Table 5 shows the effect of FLC participation on students' FK. In the counterfactual situation (*c*_2_), if the attendees did not attend, the FK score would reduce by about 0.880 (i.e., about 14.2%). In the counterfactual situation (*d*_2_), if the non-attendees had attended, their FL score would have increased by 4.184 (i.e., about 77.8%). These results indicated that the FLC attendance would significantly improve college students' FL; however, the transitional heterogeneity effect was negative, i.e., the FLC impact on attendees was significantly less than the FLC impact on the non-attendees.

The last row of Table 5 adjusts the potential heterogeneity in the sample, the results for which indicated that in the counterfactual situation (*c*_2_), the attendees' FK level would be significantly lower than the non-attendees. Finally, the counterfactual situation (*d*_2_) indicated that if the non-attendees had participated in the FLC, their FK level would have far exceeded the FK level of the actual attendees'.

### 5.5. Financial Behavior

When estimating the FLC effect on the college students' FL using the OLS, the coefficient was significantly positive, which indicated that the FLC attendance significantly and positively impacted the college students' FK (Table 6).

**Table 6:** Endogenous switching regression estimates for student's financial behavior.

	**(1)**	**(2)**	**(3)**	**(4)**
**Model**	**OLS**	**Endogenous switching regression**		
			**Attendance = 1**	**Attendance = 0**
			**Attendees**	**Non-attendees**
**Dependent variable**	**FB**	**Attendance**	**FB**	**FB**
FE	1.706^***^			
	(0.131)			
Gender	0.017	0.557^***^	−0.299	0.007
	(0.084)	(0.125)	(0.213)	(0.093)
Grade				
2^*nd*^ year	−0.255^**^	1.392^***^	−1.054^**^	−0.320^**^
	(0.115)	(0.219)	(0.455)	(0.132)
3^*rd*^ year	−0.379^***^	0.292	−0.912^*^	−0.372^**^
	(0.131)	(0.271)	(0.513)	(0.131)
4^*th*^ year	−0.590^*^	2.896^***^	−1.731^**^	−0.927
	(0.303)	(0.449)	(0.606)	(0.664)
Postgraduate	0.432	−0.049	1.392	0.503^*^
	(0.296)	(0.535)	(1.134)	(0.292)
City	0.465^***^	−0.348^**^	0.683^***^	0.281^**^
	(0.098)	(0.137)	(0.210)	(0.113)
Relationship	0.049	−0.420^***^	0.071	0.089
	(0.087)	(0.129)	(0.212)	(0.094)
OnlyChild	−0.029	−0.056	−0.041	0.051
	(0.083)	(0.122)	(0.185)	(0.089)
Major				
LiberalArts	0.157	−0.400^**^	0.193	0.151
	(0.089)	(0.137)	(0.215)	(0.095)
Engineering	0.286^**^	0.691^***^	−0.421^*^	0.344^**^
	(0.139)	(0.172)	(0.249)	(0.176)
Father's education				
HighSchoolF	0.357^***^	0.555^**^	0.096	0.263^*^
	(0.133)	(0.182)	(0.257)	(0.158)
BachelorF	0.097	0.180	0.017	0.139
	(0.148)	(0.216)	(0.302)	(0.163)
Mother's education				
HighSchoolM	−0.006	0.037	0.010	−0.025
	(0.134)	(0.183)	(0.242)	(0.155)
BachelorM	0.168	−0.486^**^	0.361	0.230
	(0.151)	(0.226)	(0.333)	(0.167)
Ranking				
Second	−0.236^*^	0.042	−0.510^*^	−0.248
	(0.140)	(0.201)	(0.285)	(0.153)
Third	−0.452^***^	0.027	−0.921^***^	−0.351^**^
	(0.131)	(0.189)	(0.282)	(0.142)
Last	−0.359^***^	−0.029	−1.081^***^	−0.150
	(0.125)	(0.181)	(0.278)	(0.136)
Family income				
Medium	0.100	0.392^**^	0.081	0.040
	(0.101)	(0.145)	(0.212)	(0.111)
High	0.377^***^	0.789^***^	−0.423	0.604^***^
	(0.138)	(0.193)	(0.257)	(0.165)
VeryHigh	0.220	0.738^***^	0.038	0.162
	(0.160)	(0.224)	(0.314)	(0.183)
Attention to FL	0.708^***^	−1.524^***^	0.609^**^	1.712^***^
	(0.124)	(0.141)	(0.290)	(0.198)
Information sources				
news		0.412^**^		
		(0.145)		
Othersmention		0.198^*^		
		(0.113)		
Media		−0.045		
		(0.124)		
Constant	4.135^***^	−1.445^***^	8.330^***^	3.171^***^
	(0.212)	(0.335)	(0.627)	(0.259)
σ_*i*_			1.324^***^	1.238^***^
			(0.115)	(0.032)
ρ_*j*_			−0.772^***^	−0.120
			(0.122)	(0.182)

*Sample size: 1,015. Robust standard errors in parentheses. σ_i_ denoted the square root of the variance of the error terms η_ji_ in the outcome Equation (3); ρ_j_ denotes the correlation coefficient between the error term ε_i_ of the selection Equation (1) and the error term η_ji_ of the outcome Equation (3)*.

*** *Significant at 1%*;

** *Significant at 5%*;

* *Significant at 10%*.

The FB level function of the attending group was significantly different (at a 1% statistical level) from the FB level function of the non-attending group [Table 6, columns (3) and (4)]. It was expected that grade, where they grew up, major, ranking, family monthly income, and attention paid to FK would be significantly associated with changes in the FB; however, grade seemed to more significantly affect the FB level of the non-attending group.

Another interesting difference between the attendees and non-attendees was the impact of household monthly income. While high household monthly income did not affect the FB of the attendees, it positively impacted the FL of the non-attendees, indicating that an increase in household monthly income would strengthen the non-attendees' FB level.

Table 7 shows the FB level of the college students under the factual and counterfactual conditions. Cells (*a*_3_) and (*b*_3_) represent the observed college students' FB observed in the sample. The attending group's expected FB score was about 6.252 points, while that of the non-attending group was about 4.907 points. However, this simple comparison may be misleading and prompt researchers to conclude that on average, the attendees' FB score would be about 1.345 (around 27.4%) higher than the FB score of the non-attendees.

**Table 7:** Average expected financial behavior (FB); treatment and heterogeneity effects.

**Sub-samples**	**Decision stage**		**Treatment effects**
	**To attend**	**Not to attend**	
College students that attended	(*a*_3_) 6.252	(*c*_3_) 3.866	TT = 2.387^***^
	(0.047)	(0.055)	(0.072)
College students that did not attend	(*d*_3_) 8.104	(*b*_3_) 4.907	TU = 3.197^***^
	(0.027)	(0.022)	(0.035)
Heterogeneity effects	BH_1_ = −1.852^***^	BH_2_ = −1.041^***^	TH = −0.810^***^
	(0.057)	(0.051)	(0.080)

*** *Significant at 1%*.

The last column in Table 7 shows the influence of FLC participation on the students' FB. In the counterfactual situation (*c*_3_), if the attendees had not attended, the FB score would have been reduced by about 2.387 (about 38.2%). In the counterfactual situation (*d*_3_), if the non-attendees had attended, their FL score would have increased by 3.197 (about 65.1%). These results indicated that the FLC attendance would significantly improve college students' FB; however, the transitional heterogeneity effect was negative, i.e., the FLC impact on the attendees was significantly less than that of the non-attendees.

The last row of Table 7 adjusts the potential heterogeneity in the sample, the results from which indicated that in the counterfactual situation (*c*_3_), the attendees' FB levels were significantly lower than the non-attendees. Finally, counterfactual situation (*d*_3_) indicated that if non-attendees had participated in the FLC, their FB levels would have far exceeded those of the actual attendees'.

### 5.6. Financial Attitude

The OLS estimates indicated that attending an FLC significantly and positively impacted the college students' FA (Table 8).

**Table 8:** Endogenous switching regression estimates for student's financial attitude (FA).

	**(1)**	**(2)**	**(3)**	**(4)**
**Model**	**OLS**	**Endogenous switching regression**		
			**Attendance = 1**	**Attendance = 0**
			**Attendees**	**Non-attendees**
**Dependent variable**	**FA**	**Attendance**	**FA**	**FA**
FE	0.441^***^			
	(0.051)			
Gender	−0.069^**^	0.608^***^	−0.022	−0.068^*^
	(0.033)	(0.126)	(0.088)	(0.037)
Grade				
2^*nd*^ year	0.019	1.396^***^	−0.428^**^	0.111^**^
	(0.045)	(0.217)	(0.189)	(0.052)
3^*rd*^ year	0.006	0.237	−0.613^**^	0.062
	(0.051)	(0.266)	(0.229)	(0.053)
4^*th*^ year	−0.072	2.925^***^	−0.650^**^	0.452^*^
	(0.118)	(0.440)	(0.246)	(0.252)
Postgraduate	0.096	−0.278	1.519^**^	0.035
	(0.116)	(0.538)	(0.522)	(0.118)
City	0.129^***^	−0.364^**^	0.375^***^	0.037
	(0.038)	(0.137)	(0.093)	(0.045)
Relationship	0.013	−0.430^***^	0.131	−0.032
	(0.034)	(0.129)	(0.090)	(0.038)
OnlyChild	−0.033	−0.154	−0.002	−0.028
	(0.032)	(0.121)	(0.080)	(0.036)
Major				
LiberalArts	0.042	−0.434^**^	0.056	0.025
	(0.035)	(0.137)	(0.093)	(0.038)
Engineering	−0.035	0.748^***^	−0.107	0.026
	(0.054)	(0.170)	(0.107)	(0.069)
Father's education				
HighSchoolF	0.092^*^	0.561^**^	0.052	0.101
	(0.052)	(0.178)	(0.111)	(0.063)
BachelorF	0.082	0.244	0.032	0.110^*^
	(0.058)	(0.214)	(0.131)	(0.065)
Mother's education				
HighSchoolM	−0.132^**^	0.039	−0.179^*^	−0.055
	(0.052)	(0.178)	(0.105)	(0.062)
BachelorM	−0.128^**^	−0.551^**^	−0.097	−0.136^**^
	(0.059)	(0.222)	(0.141)	(0.067)
Ranking				
Second	−0.050	−0.007	0.007	−0.096
	(0.055)	(0.201)	(0.126)	(0.061)
Third	0.002	−0.035	0.056	0.000
	(0.051)	(0.187)	(0.124)	(0.057)
Last	−0.102^**^	−0.117	−0.093	−0.078
	(0.049)	(0.180)	(0.123)	(0.054)
Family income				
Medium	−0.001	0.459^***^	−0.084	0.023
	(0.039)	(0.144)	(0.093)	(0.044)
High	−0.087	0.773^***^	−0.165	−0.038
	(0.054)	(0.191)	(0.111)	(0.066)
VeryHigh	0.025	0.658^**^	−0.168	0.102
	(0.063)	(0.223)	(0.140)	(0.073)
Attention to FL	−0.096^**^	−1.550^***^	0.208^*^	−0.211^**^
	(0.048)	(0.137)	(0.117)	(0.076)
Information sources				
news		0.489^***^		
		(0.146)		
Othersmention		0.172		
		(0.113)		
Media		−0.123		
		(0.116)		
Constant	2.972^***^	−1.356^***^	3.862^***^	3.122^***^
	(0.083)	(0.336)	(0.265)	(0.102)
σ_*i*_			0.563^***^	0.505^***^
			(0.045)	(0.016)
ρ_*j*_			−0.682^***^	0.606^***^
			(0.130)	(0.137)

*Sample size: 1,015. Robust standard errors in parentheses. σ_i_ denoted the square-root of the variance of the error terms η_ji_ in the outcome Equation (3); ρ_j_ denotes the correlation coefficient between the error term ε_i_ of the selection equation Equation (1) and the error term η_ji_ of the outcome Equation (3)*.

*** *Significant at 1%*;

** *significant at 5%*;

* *significant at 10%*.

The FA level function of the attending group was significantly different (at a 1% statistical level) from the FA level function of the non-attending groups [Table 8, columns (3) and (4)]. It was expected that gender, hometown, father's education, mother's education, grade ranking, and attention to FK would be significantly associated with changes in the FA; however, mainly gender, grade, and mother's education more significantly affected the FA level of the non-attending group.

Table 9 shows the college students' FA levels under the factual and counterfactual conditions. Cells (*a*_4_) and (*b*_4_) represent the observed college students' FA observed in the sample. The attending group's expected FB score was about 3.324 points, while that of the non-attending group was about 2.887 points. However, this simple comparison may be misleading and prompt researchers to conclude that the average attendees' FA would be about 0.437 (around 15.2%) higher than that of the non-attendees.

**Table 9:** Average expected FA; treatment and heterogeneity effects.

**Sub-samples**	**Decision stage**		**Treatment effects**
	**To attend**	**Not to attend**	
College students that attended	(*a*_4_) 3.324	(*c*_4_) 3.334	TT = −0.010
	(0.019)	(0.011)	(0.022)
College students that did not attend	(*d*_4_) 4.013	(*b*_4_) 2.887	TU = 1.126^***^
	(0.014)	(0.004)	(0.014)
Heterogeneity effects	BH_1_ = −0.688^***^	BH_2_ = 0.447^***^	TH = −1.135^***^
	(0.029)	(0.009)	(0.039)

*** *Significant at 1%*.

The last column in Table 9 shows the influence of FLC participation on the students' FA. In the counterfactual situation (*c*_3_), if the attendees had not attended, there would have been a little variance in the FA score (only a slight increase of about 0.010, or 0.3%, which was not statistically significant). In the counterfactual situation (*d*_4_), if the non-attendees had attended, their FL score would have increased by 1.126 (about 39.0%). These results indicate that the FLC attendance would significantly improve college students' FA; however, the transitional heterogeneity effect was negative, i.e., the FLC impact on the attendees was significantly less than the FLC impact on the non-attendees.

The last row of Table 9 adjusts the potential heterogeneity in the sample, the results from which indicated that in the counterfactual situation (*c*_4_), the attendees' FA levels were significantly lower than the non-attendees' FA levels. Finally, the counterfactual situation (*d*_4_) indicates that if the non-attendees had participated in the FLC, their FA levels would have far exceeded those of the actual attendees.

### 5.7. Robustness Check

Alternative specifications were conducted to ensure the validity of the results. One consideration was that there may have been some measurement errors in the FL test. Therefore, a novel FL measure based on a hybrid IRT was applied. Similar to factor analysis, which considers the weight of an FK (FB or FA) in FL, IRT estimates the difficulty of an FK (FB or FA) and places a larger weight on the FL of an individual who exhibits more difficult FK (FB or FA) (Kunovskaya et al., 2014), hybrid IRT is more suitable for a questionnaire when the questions on FB and FA have no correct answers (Chiang, 2020)^7^. The result shows that as for the unweighted FL measure, the weighted measure was also affected by factors such as grade, where the respondents grew up, major, father's education, grade ranking, family monthly income, and attention to FK^8^. Accordingly, the hybrid IRT scoring method assigned greater weight to the FL of a student who exhibited more difficult FK (FB or FA) to help examine the sensitivity of the estimates to the type of questions included in the FL index. It was interesting that this alternative FL index was again positive and statistically significant; however, the magnitudes of the ESR coefficient (Table 10) and the OLS coefficient were much larger than shown in Table 3.

**Table 10:** Average expected FL (weighted); treatment and heterogeneity effects.

**Sub-samples**	**Decision stage**		**Treatment effects**
	**To attend**	**Not to attend**	
College students that attended	(*a*_1_) 1.122	(*c*_1_) −0.517	TT = 1.639^***^
	(0.265)	(0.122)	(0.018)
College students that did not attend	(*d*_1_) 1.610	(*b*_1_) −0.309	TU = 1.919^***^
	(0.337)	(0.126)	(0.011)
Heterogeneity effects	BH_1_ = −0.487^***^	BH_2_ = −0.208 ^***^	TH = −0.279^***^
	(−0.536)	(−0.227)	(0.021)

*** *Significant at 1%*.

Because ESR results can be sensitive due to model assumptions (such as the selection of instrumental variables), the PSM approach was also applied to check the robustness of the ESR results (Table 11). Three matching algorithms were implemented: kernel matching (KM), radius matching (RM), and nearest-neighbor matching (NNM). Regardless of the matching algorithm was used in the estimation, the PSM estimates showed that the FLC attendance significantly improved the students' FL skills (Table 11, the sum scoring method), with the increase in FL ranging from 3.182 to 3.356 points (25.1–26.8%).

**Table 11:** PSM estimates of the FLC impacts.

**Matching algorithm**	**Scoring method**	**Means of outcome variables**		**ATT difference**
		**Attendees**	**Non-attendees**	
Kernel matching	Sum scoring	15.858	12.653	3.205^***^
	Hybrid-IRT Sum scoring	1.131	−0.412	1.543^***^
Radius matching	Sum scoring	15.858	12.676	3.182^***^
	Hybrid-IRT Sum scoring	1.131	−0.405	1.537^***^
Nearest neighbor matching	Sum scoring	15.858	12.502	3.356^***^
	Hybrid-IRT Sum scoring	1.131	−0.411	1.542^***^

*Sample size: 1,015. Absolute values of z-statistics in parentheses*.

*** *Indicates the coefficient is different from zero at the 1% level*.

Different FL measuring methods were considered during the PSM process (Table 11, Hybrid-IRT scoring method). Once again, the FL measured in this alternative way was again positive and statistically significant, i.e., the FLC elevated the students' FL, which suggested that the FLC had a positive impact on the college students' FL and the relationship did not depend on a specific FL measure.

## 6. Conclusion

Financial education has always been a heated topic in policy forums. Although many are delighted about the prospects of improving an individual's financial capabilities through these FE programs, there has been little robust evidence supporting the FE program's effectiveness. However, due to the many national FE programs, which have been focused on younger people and included recommendations to pioneer financial curricula in schools, there has been a significant rise in evaluation studies.

This study gave a timely and complete sketch of the impact of possible school-based FE programs. First, it examined the driving forces behind college students' decision to attend an FLC. Second, to exclude endogeneity and heterogeneity, an ESR was employed to evaluate the FLC effects on college student FL.

The analysis of the attendance determinants highlighted very interesting results. Apart from student's grade and family monthly income, which were found to have a positive impact on the probability of attendance, almost all of the other factors' impact indicated that students with lower self-perceived FL levels would more likely to choose to attend the FLC; however, those students with higher self-perceived FL were found to prefer not to choose to attend an FLC.

Three main conclusions were drawn from the results of the impacts of the FLC attendance on FL. First, the group of attendees had systematically different characteristics than the group of the non-attendees. Because these disparities indicated that there were variations between the two groups, an OLS model that included a dummy variable for FLC attendance or non-attendance could not be taken into account.

Second, FLC attendance was found to contribute to sizeable and robust impacts on the college student's FL, which comprised FK, FA, and FB. When this result was analyzed for the two different “attendee” and “non-attendee” student groups, interesting patterns emerged. Students who did not attend tended to be found to benefit more than the attendees in the counterfactual case, and the “non-attendees” were found to have some characteristics (e.g., unobserved skills) that made them more financially literate even without attendance, which also possibly explained the third finding. Interestingly, it was also found that the attendance impact on the FLC was smaller for the attendees than for the non-attendees in the counterfactual case that they attended. It seemed, therefore, that while both college students groups would benefit from the FLC attendance, the students that did not attend would benefit the most from attendance. This beneficial attendance effect was found to be large. If the students that did not attend had attended, they would have benefited much more than the students that actually attended. However, as the FL of the attendees was found to sharply decrease if they had not attended, the FLC seemed to be particularly important for the attendees and helped them to close the FL gap with the more financially literate students.

The impact comparison of the FLC estimated using the different methods had verified that the FLC has a significant positive impact on enhancing the college students' FL. Regardless of which method was applied to measure the FL level of the college students, the FLC was still found to significantly improve their FL. The inclusive effect was unique and made a strong case for the universalization of the FLC at college. Students with lower FL took part in the training and those with higher FL did not take part, with the results indicating that students who had not participated performed better, which tended to indicate that the training is more effective for people with some prior knowledge. The ESR analysis that accounted for the student characteristics, the baseline FK heterogeneity, and the sample selection bias yielded a surprising and very promising result: the FLC for college students allows all students to enhance their measured FL levels. These results highlighted that irrespective of the unobservable characteristics, it was important to attend an FLC to improve student FL and proved that the FLC should be compulsory to improve overall student FL skills.

## 7. Limitations

Regardless of the positive results, this study had some limitations. The main limitation was that the control group did not complete the post-curriculum survey and it was presumed that the student's FL would not change over a relatively short time. However, this could have provided an estimate of test-retest reliability (control group only, since they were not exposed to the FLC). As it is possible that the FL changed over time as a function of college attendance and other confounding factors (such as maturation), this question remains unanswered but is a possible counterfactual. Therefore, it is recommended that further study be carried out to confirm the assumptions that students' FL does not change over a certain period. Therefore, the study results should be considered tentative.

Due to the class number limitations, the study conducted two FLC rounds. Moreover, given that the FLC was delivered over two rounds, this may have resulted in a group or clustering (dependency) effect on the outcomes. Readers should, therefore, adopt the present findings and conclusions with caution. Although there were some limitations, these do not weaken the significance of the findings, which provide a framework for researchers for further research.

## Data Availability Statement

The raw data supporting the conclusions of this article will be made available by the authors, without undue reservation.

## Author Contributions

JX contributed to the conception of the study. XL, ZH, YN, QY, YL, and JX contributed significantly to the data sources and manuscript preparation. XT performed the data analyses and wrote the manuscript. ZH, YN, QY, YL, and JX helped perform the analysis with constructive discussions.

## Funding

The study is supported by the National Natural Science Foundation of China (grant nos. 72072122 and 71572120).

## Conflict of Interest

The authors declare that the research was conducted in the absence of any commercial or financial relationships that could be construed as a potential conflict of interest.

## Publisher's Note

All claims expressed in this article are solely those of the authors and do not necessarily represent those of their affiliated organizations, or those of the publisher, the editors and the reviewers. Any product that may be evaluated in this article, or claim that may be made by its manufacturer, is not guaranteed or endorsed by the publisher.

## Footnotes

^1^The frame was developed by a team of experts organized by the China Financial Literacy Education Collaborative Innovation Center during the year of 2016–2018.

^2^ https://www.icourse163.org/course/SCU-1002879002

^3^Considering the teaching target (all college students from all majors) of the FLC and the teaching goals (to foster students' financial literacy and financial decision-making ability), only the second chapter (the value concepts of financial management) of the online course 'financial management' was a compulsory study requirements. Because all the remaining chapters are more inclined to financial analysis, some basic knowledge of financial management is required, which is more suitable for the finance majors.

^4^This intermediate test was only a periodic test for online financial knowledge learning. The test results were included in the final course assessment results and affected the student's grade point. The purpose of the intermediate test was to urge students to earnestly study financial knowledge and was not directly related to the follow-up effect evaluation of the FLC.

^5^Survey methodology and sampling techniques: An online survey technique was employed for the data collection. No student identity information was gathered on the survey. The first email message was sent to 3,000 students who were randomly selected from the entire student population, including graduate students. The email message asked students to visit a website in order to complete the online survey.

^6^The score is based on a derived variable, created from the responses to two questions (FB5-1 and FB5-2). 1 point if personally or jointly responsible for money management (FB5-1) and household has a budget (FB5-2). 0 in all other cases.

^7^Instead of just adding the items up and focusing on the test as unit of analysis, hybrid IRT focuses on each item as an analysis unit, which provides a more precise evaluation of the structure of the multidimensional constructs.

^8^The table of the result was not shown here due to the space limitation, but once upon request, it would be made available by the corresponding authors.
