# Supplementary material for: Is It Necessary to Launch a School-Based Financial Literacy Curriculum? Evidence From China
Source: Front Psychol. 2022 Apr 19;13:846382. doi: 10.3389/fpsyg.2022.846382 (PMC9063735; doi:10.3389/fpsyg.2022.846382)
Supplement: Supplementary file 1 [file Data_Sheet_1.pdf]

frontiers

# *Supplementary Material*

## APPENDIX A.

Table S1: Variables definition

| Variable name | Definition |
|---|---|
| *Dependent variables* | |
| Adaptation | =1 if a college student attended to the FLC, 0 otherwise |
| FL | student financial literacy |
| FK | student financial knowledge |
| FB | student financial behavior |
| FA | student financial attitude |
| *Explanatory variables* | |
| *Gender* | = 1 if a student is female, 0 otherwise |
| *Grade* | |
| $2^{nd}$ year | = 1 if a student is a $2^{nd}$ year undergraduate, 0 otherwise |
| $3^{rd}$ year | = 1 if a student is a $3^{rd}$ year undergraduate, 0 otherwise |
| $4^{th}$ year | = 1 if a student is a $4^{th}$ year undergraduate, 0 otherwise |
| Postgraduate | = 1 if a student is a postgraduate, 0 otherwise |
| *City* | = 1 if a student grew up in a town, 0 otherwise |
| *Relationship* | = 1 if a student is in a relationship, 0 otherwise |
| *OnlyChild* | = 1 if a student is the only child in his/her family, 0 otherwise |
| *Major* | |
| LiberalArts | = 1 if a student majors in liberal arts, 0 otherwise |
| Engineering | = 1 if a student majors in engineering, 0 otherwise |
| *Father's education* | |
| HighSchoolF | = 1 if a student's father has a high school diploma, 0 otherwise |
| BachelorF | = 1 if a student's father has a bachelor degree or above, 0 otherwise |
| *Mother's education* | |
| HighSchoolM | = 1 if a student's mother has a high school diploma, 0 otherwise |
| BachelorM | = 1 if a student's mother has a bachelor degree or above, 0 otherwise |
| *Ranking* | |
| Second | = 1 if a student's general grade ranked among 11%–20% in his/her class, 0 otherwise |
| Third | = 1 if a student's general grade ranked among 21%–50% in his/her class, 0 otherwise |
| Last | = 1 if a student's general grade ranked among 51%–100% in his/her class, 0 otherwise |
| *Family income* | |
| Medium | = 1 if a student's family income ranges from 5,000–1000 CNY, 0 otherwise |
| High | = 1 if a student's family income ranges from 10,000–20,000 CNY, 0 otherwise |
| VeryHigh | = 1 if a student's family income ranges from 10,000–20,000 CNY, 0 otherwise |
| *Attention to FL* | = 1 if a student pay attention to financial knowledge in the daily life, 0 otherwise |

Table 13 Continued

| Variable name | Definition |
|---|---|
| *Information sources* | |
| News | = 1 if a student got the FLC information from college daily news, 0 otherwise |
| Othersmention | = 1 if a student got the FLC information from others people's mention, 0 otherwise |
| Media | = 1 if a student got the FLC information from social media, 0 otherwise |

**Table S2.** A summary table of score information

| Group of students | Obs | Mean | Std.Dev. | Min | Max |
|---|---|---|---|---|---|
| Pre-FLC not attend | 796 | 13.178 | 1.942 | 6.333 | 17.667 |
| Pre-FLC attend | 219 | 12.618 | 2.681 | 4.333 | 18.000 |
| Post-FLC attend | 219 | 15.790 | 2.096 | 10.333 | 21.000 |

## APPENDIX B.COLLEGE STUDENTS' FL MEASUREMENT

(FK1 *Time value of money*) Now imagine that you have to wait for one year to get 1,000 yuan, and the inflation stays at 5 percent. In one year's time will you be able to buy:

(a)More than 1000 yuan; (b)Exactly 1000 yuan; **(c)Less than 1000 yuan**; (d)It depends on the types of things that I want to buy; (e)Don't know; (f)Refused.

(FK2 *Interest on loan*) You lend 500 yuan to your classmate, and he/her will pay you 500 yuan back three months later. Assuming that the bank's annual loan interest rate is 4.35%, how much interest has he paid on this loan?

(a)5.4375 yuan; (b)21.75 yuan; **(c)0**; (d)Don't know; (e)Refused.

**Table S3.** Parameter estimates-Test on the validity of the selection instruments

| VARIABLES | (1)<br>Model 1 | (2)<br>Model 2 | (3)<br>Model 3 | (4)<br>Model 4 | (5)<br>Model 5 |
|---|---|---|---|---|---|
| Information sources | | | | | |
| News | 0.334*** | −0.0261 | −0.0756 | 0.0609 | −0.0113 |
| | (0.115) | (0.165) | (0.0988) | (0.123) | (0.0416) |
| Othersmention | 0.201** | 0.193 | 0.129 | 0.0360 | 0.0282 |
| | (0.0919) | (0.142) | (0.0860) | (0.101) | (0.0359) |
| Media | −0.0758 | −0.00543 | 0.00330 | 0.00305 | −0.0118 |
| | (0.0928) | (0.146) | (0.0888) | (0.105) | (0.0375) |
| Constant | −1.124*** | 13.10*** | 5.371*** | 4.840*** | 2.887*** |
| | (0.119) | (0.161) | (0.102) | (0.121) | (0.0422) |
| Wald test | $\chi^2$= 13.65*** | F-stat.= 0.64 | F-stat.= 0.91 | F-stat.= 0.14 | F-stat.= 0.24 |
| Observations | 1,015 | 796 | 796 | 796 | 796 |
| $R$-squared | | 0.002 | 0.004 | 0.001 | 0.001 |

Model 1: Probit model; Model 2-5: ordinary least squares. Standard errors in parentheses. *** Significant at 1%; ** Significant at 5%. Parameters for all the other variables are not reported.

(FK3 *Simple interest*) Suppose you put 100 yuan into a savings account with a guaranteed interest rate of 2% per year. You don't make any further payments into this account and you don't withdraw any money. How much money would be in the account at the end of the first year, once the interest payment is made?

**(a)102 yuan**; (b)120 yuan; (c)Do not know; (d)Refused.

(FK4 *Compound interest*) Suppose you put 1,000 yuan in the bank with an annual interest rate of 2%. What is the balance of your account at the end of five years?

**(a)Over 1100 yuan**; (b)Exactly 1100 yuan; (c)Less than 1100 yuan; (d)Or is it impossible to tell from the information given; (e)Don't know; (f)Refused.

(FK5 *Risk and Return*) An investment with a high return is likely to be high risk

**(a)Correct**; (b)Wrong; (c)Don't know; (d)Refused.

(FK6 *Definition of Inflation*) High inflation means that the cost of living is increasing rapidly

**(a)Correct**; (b)Wrong; (c)Don't know; (d)Refused.

(FK7 *diversified investment*) It is usually possible to reduce the risk of investing in the stock market by buying a wide range of stocks and shares.

**(a)Correct**; (b)Wrong; (c)Don't know; (d)Refused.

(FK8 *Bond Knowledge*) If interest rates fall, the price of bonds would

(a)Decline; **(b)rise**; (c)Don't know; (d)Refused.

(FA1: *Live for today*) I tend to live for today and let tomorrow take care of itself:

(a) Completely agree(1); (b) Agree(2); (c) Undecided(3); (d) Disagree(4); (e) Completely disagree(5).

(FA2 *spend than save*) I find it more satisfying to spend money than to save it for the long term:

(a) Completely agree(1); (b) Agree(2); (c) Undecided(3); (d) Disagree(4); (e) Completely disagree(5).

(FA3 *money is there to be spent*) Money is there to be spent.

(a) Completely agree(1); (b) Agree(2); (c) Undecided(3); (d) Disagree(4); (e) Completely disagree(5).

(FB1 *Affordability*) Before I buy something I carefully consider whether I can afford it:

(a) **Completely agree**; (b) **Agree**; (c) Neutral; (d) Disagree; (e) Completely disagree.

(FB2 *bill payment*) I pay my bills on time:

(a) **Completely agree**; (b) **Agree**; (c) Neutral; (d) Disagree; (e) Completely disagree.

(FB3 *Financial Monitoring*) I keep a close personal watch on my financial affairs:

(a) **Completely agree**; (b) **Agree**; (c) Neutral; (d) Disagree; (e) Completely disagree.

(FB4 *Long-term planning*) I set long term financial goals and strive to achieve them:

(a) **Completely agree**; (b) **Agree**; (c) Neutral; (d) Disagree; (e) Completely disagree.

(FB5-1 *personal budget*) Do you have a budget for daily consumption?

(a)**Yes**; (b)No; (c)Don't know; (d)Refused.

(FB5-2 *Decision-making*) Will you participate in the financial decision-making in your family?

(a)**Yes**; (b)No; (c)Don't know; (d)Refused.

(FB6 *Actively savings*) In the past 12 months have you been [individually] saving money, whether or not you still have the money?

(a) **Yes**(1 point); (b) No; (c) Don't know; (d) Refused.

(FB7 *Choosing financial products*) Which of the following statements best describes your choice about choosing financial products?

(a)**I considered several options from different companies before making my decision**(1 point); (b)**I considered the various options from one company**; (1 point) (c)**I didn't consider any other options at all** ;(1 point) (d)**I looked around but there were no other options to consider**; (1 point) (e)Don't know; (f)Not applicable; (g)Refused.

(FB8 *Making ends meet*) What did you do to make ends meet the last time this happened?

(1) **Cut back on spending, spend less, do without**(1 point); (2) **Sell something that you own**(1 point); (3) **Do some part-time jobs to make money**(1 point); (4) Ask help from parents; (5) Borrowing from classmates or friends; (6) Online lending; (7) Don't know; (8) Refused.
